# Periscope of Dermatology

**Published:** 1897-05-29

**Authors:** 


### Periscope of Dermatology.

(*Continued from page* 132.)

**Herpes Zoster.**—Mahon[24] records two cases of the occurrence of herpes zoster below the knee. In one case a woman, aged 69 years, developed patches of vesicles on the back of the leg, on the dorsum of the foot, and on the outer side of the little toe. Other patches appeared over the sacrum, vulva, and thighs. In the second case, also a woman, aged 76 years, patches of herpes occurred on the front and outer sides of the leg, and just below the ankle. Other patches occurred over the sacrum, buttocks, and thigh. The illness was attributed to getting a chill by walking barefoot through wet grass.

Symons Eccles[25] records the case of a man, aged 25 years, who developed patches of herpes over both shins about four days after an attack of intercostal herpes. It occurred after exposure to cold during a long night journey by train.

Crisp[26] records two cases of herpes occurring below the knee; both patients were females, and no definite cause of the disease was found. The eruption in one case was preceded by severe pain for an unusually long time.

Browne[27] also records the case of a woman, aged 72, who, after taking arsenic, developed herpes on the leg and foot. The accompanying pain did not entirely disappear till five months after the onset of the attack.

Axford[28] relates the case of a lad, aged 20, who developed groups of vesicles on the anterior and outer aspects of the right leg just above the ankle joint, corresponding to the distributions of the ultimate divisions of the lumbo-sacral cord. A week before the eruption appeared the patient had an abrasion of the ankle from friction of a boot.

**X-Ray Dermatitis.**—Crocker[29] describes the case of a lad, aged 16 years, who developed dermatitis of his abdomen after an attempt had been made to radiograph his spine. A Crook's tube was placed five inches from the epigastrium, and an exposure of one hour was made. The next day the skin felt irritable, and was of a deep red colour in the area subjected to the rays. The irritability increased, and on the ninth day vesicles appeared on what was then a raised red patch. The whole of the epidermis subsequently separated, but the hairs remained. Crocker refers to the other recorded cases of dermatitis due to X-rays, and considers that, though these rays may produce dermatitis like severe sunburn, and even ulceration, these ill-effects are only produced when the exposure is very prolonged, and the Crooke's tube is placed very close to the skin. No ill-effects from the exposure required for an ordinary radiograph need be feared. He suggests that red pigments might be employed by workers in these rays as a prophylactic against their ill-effects.

Gilchrist[30] refers to 23 cases of dermatitis caused by X-rays, and gives a minute record of the case of a man aged 32 years, who developed dermatitis after exposing his arm during three weeks for four hours daily. The skin became red and puffed up and ached. The epidermis afterwards exfoliated. On taking X-ray photographs of the hands afterwards, the bones of the affected hand were found to be thickened. Microscopic examination of the affected skin only showed chronic inflammatory changes. Recovery was very gradual. He suggests that in order to avoid these ill-effects the palmer surface of the hand should be exposed to the rays since it is protected by a much thicker horny layer.

King[31] records the case of a man who experimented for about six hours daily with the X-rays. After about six weeks the right hand became swollen, and blisters appeared on its dorsal aspect. Picric acid allayed the pain and rendered the hand less susceptible, so the patient continued his work. After a time the nails exfoliated, and the hair on the face fell out. He thought, also, that his sight was impaired.

**Tuberculosis of Skin.**—Moyniham[32] records a case of tuberculosis verrucosa of the skin which occurred in a girl aged 20. Three months previously she had cut her hand, and had washed some handkerchiefs of a phthisical patient. The wound refused to heal, and some inflammation extended round the wound. The patch of disease was removed widely, and the wound healed soundly. Microscopically the epidermis was thickened, and the underlying round cell-infiltration resembled tuberculous infiltration. Giant cells were also seen.

Darier[33] points out that the group of affections comprising acne scrofulosorum, folliclis, hidrosadenitis destuens, granuloma innominata, and disseminated lupus erythematosus are related to each other and to tuberculosis, since they are all invariably characterised by successive eruptions, beginning in the form of papules or introdermic indolent nodules that become capped by a pustule which bursts or dries in a crust. Their evolution, too, is slow. They are frequently met with in patients suffering from pulmonary or glandular tuberculosis.

Dent[34] records the case of a girl aged 19 years who had suffered from lupus in the limbs for 15 years. She had been treated six years ago with tuberculin injections, and undoubted improvement had followed since that time. Three sisters of the patient also suffered from lupus, and it was suggested that they had contracted the disease by sleeping in the same bed as the patient.

[24] Brit. Med. Journ., Nov. 7, 1896, p. 1,388. [25] Ditto, Jan. 2, 1897, p. 14. [26] Ditto, ditto. [27] Ditto, ditto. [28] Ditto, ditto. [29] Jan. 2, 1897, p. 68. [30] Johns Hopkins Bull., Feb., 1897, p. 17. [31] Brit. Journ. of Derm., Feb., 1897, p. 82. [32] Brit. Med. Journ., Jan. 9, 1897, p. 77. [33] Med. Week Dec. 26, 1896, p. 620. [34] Brit. Journ. of Derm., Dec., 1896, p. 478.

